# 磁流体热疗对体外人肺癌细胞A549的影响

**DOI:** 10.3779/j.issn.1009-3419.2011.03.01

**Published:** 2011-03-20

**Authors:** 国卿 王, 浒 李, 润磊 胡, 贤福 柯, 东山 魏, 文 孟

**Affiliations:** 310006 杭州，杭州市第一人民医院胸外科 Department of Thoracic Surgery, Hangzhou First People's Hospital, Hangzhou 310006, China

**Keywords:** 肺肿瘤, 凋亡, 体外, Lung neoplasms, Apoptosis, *In vitro*

## Abstract

**背景与目的:**

磁流体热疗是将纳米技术和热疗相结合的一种新兴热疗方法，具有高度的靶向性和特异性，已成为目前研究的热点。本研究旨在探讨Fe_3_O_4_纳米磁流体热疗在体外对人肺癌A549细胞的影响。

**方法:**

在体外将不同浓度的Fe_3_O_4_纳米磁流体和人肺癌A549细胞共培养，在交变磁场中作用30 min，采用MTT法细胞计数、流式细胞术、倒置显微镜、透射电镜等方法观察磁流体热疗对人肺癌A549细胞活细胞数的光密度值、杀伤率、细胞形态、细胞周期及凋亡情况的影响。

**结果:**

磁流体热疗后人肺癌A549细胞增殖受到明显抑制，活细胞数的光密度值下降；杀伤率（cytotoxity index, CI）增加；细胞凋亡率逐渐增强；细胞周期停滞于S期，S期细胞和G_2_期增加，与磁流体浓度呈明显依赖关系。电镜观察磁流体热疗后的肺癌细胞呈凋亡样改变，高温时呈坏死样改变。

**结论:**

磁流体热疗能明显抑制人肺癌A549细胞的增殖，诱导凋亡，阻滞细胞于S期。

## Abstract (en)

**Background and objective:**

Magnetic fluid hyperthermia (MFH) is a method of heat therapy using nanometer techniques and hyperthermia. It has the advantage of high specificity of targeting. The aim of this study is to detect the effects of MFH induced by an alternating magnetic field on human being carcinoma A549 cells *in vitro*.

**Methods:**

A human adenocarcinoma cell line A549 was cultured with various concentrations of ferroferric oxide (Fe_3_O_4_) magnetic fluid (1.5-6.0) mg/mL and exposed to an alternative magnetic field (AMF) for 30 min. And then the optical density (OD) of viable cell, cytotocixity index, growth curve of cells, morphologic changes of cell, cell cycle and aposptosis were measured.

**Results:**

The proliferation of the A549 cells were remarkably inhibited, the OD value of viable cells decreased and cytotoxity index (CI) increased; Apoptosis of the A549 cells were observed to have cell shrinkage, chromatin condensation, margination, unclear fragmentation and intact cell membrane by light and electron microscopy; The cells were inhibited in the stage S.

**Conclusion:**

MFH induced by AMF could inhibit the proliferation, which promotes apoptosis and arrest at S stage of the A549 cells.

肺癌是严重危害人类生命健康的常见疾病，发病率高，预后较差。传统的手术、化疗以及放疗可使部分患者受益。但是大部分患者在确诊时多失去手术机会^[1]^。因此，临床上迫切需要采取另一种有效的办法来治疗肺癌。

肿瘤热疗（或称加温治疗）是近年来发展较快的一种治疗方法，传统的热疗方法均有靶向性差的特点，常导致周围正常组织损伤，因此应用比较局限。磁流体热疗（magnetic fluid hyperthernia, MFH）是将纳米技术和热疗相结合的一种新兴热疗方法。Jordan等^[2]^率先开展了此方面的研究。将纳米级的磁流体直接注射到肿瘤内部，在交变磁场的作用下磁流体通过Neel机制升温至有效的温度^[3]^，从而杀死肿瘤细胞，而周围正常组织没有磁流体的分布，不升温或升温不明显，因此具有高度的靶向性和特异性。

近年来，国内外学者在此方面做了部分研究工作，研究主要集中在实验研究阶段，涉及到的肿瘤主要有黑色素瘤、肝癌、乳腺癌、神经胶质瘤等^[4-7]^。研究证实，磁流体热疗可以抑制肿瘤的生长，延长生存期，但是涉及到人肺癌的研究未见报道。为此，本研究从体外研究Fe_3_O_4_磁流体在交变磁场下对人肺癌细胞A549的作用及其作用机制，从而为体内抗肿瘤实验提供依据。

## 1 材料与方法

### 1.1 主要材料

#### 1.1.1 主要试剂

MTT、DMSO、胰蛋白酶、RPMI-1640均由Sigma公司提供。

#### 1.1.2 细胞株

人肺癌细胞株A549由杭州昊天生物有限公司提供，用含10%胎牛血清RPMI-1640培养液。

#### 1.1.3 磁流体

纳米级Fe_3_O_4_颗粒，采用化学共沉淀法制成胶体混悬液，呈黑色。样品粒径范围为10 nm-40 nm，磁饱和强度是360 GS。样品使用前均以超声波处理5 min，使Fe_3_O_4_颗粒分布更均匀（图 1）。

**1:**
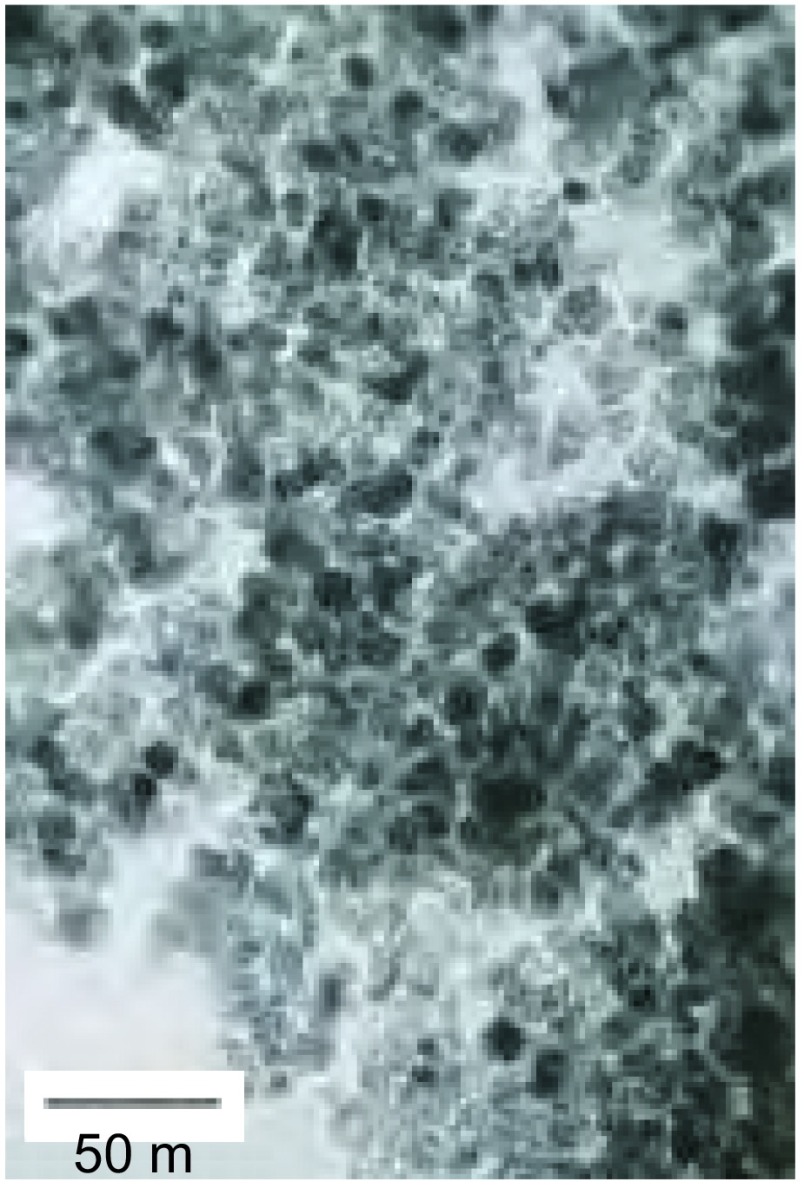
磁流体粒子的电镜照片。照片显示粒子直径在10 nm-40 nm之间（×200万） SEM images of magnetic fluid particle. The picture showed that the diameter of the particle was about 10 nm-40 nm (×2, 000, 000)

#### 1.1.4 主要仪器设备

高频感应加热机（型号：SP-04AC 4 KW）：深圳市双平电源技术有限公司提供，频率为100 KHz-250 KHz，感应线圈，由4匝直径为4 mm的铜管平行绕成内径为3 cm、长为4 cm的线圈，铜管内通循环水。流式细胞仪（型号EPICS XL型）：美国Coulter公司产品。透射电镜（JEM-1230型透射）：日本JEOL公司产品。

### 1.2 实验方法

#### 1.2.1 细胞计数法观察各组人肺癌细胞的生长曲线

将1×10^5^/mL的细胞悬液接种于10 cm^2^培养瓶中，按分组A、B、C、D、E顺序加入含有不同浓度磁流体的培养液，使终浓度分别为0 mg/mL、1.5 mg/mL、3.0 mg/mL、4.5 mg/mL、6.0 mg/mL，每瓶3 mL，每个时间点设置3瓶，交变磁场作用30 min，参照文献^[8]^绘制生长曲线。

#### 1.2.2 MTT法观察人肺癌活细胞的光密度值及杀伤率

取对数生长的人肺癌细胞A549，将细胞调成1×10^5^/mL的浓度，设置对照组（A组）和实验组，实验组按B、C、D、E顺序加入含Fe_3_O_4_磁性纳米粒的RPMI-1640培养液，每瓶3 mL，使各组终浓度分别为1.5 mg/mL、3.0 mg/mL、4.5 mg/mL、6.0 mg/mL，以没有加磁流体的RPMI-1640培养液作为A组，交变磁场作用30 min，参照文献^[9]^行MTT实验。用酶标仪590 nm处测量光密度值（optical density, OD），取均值，计算细胞生长抑制率[即杀伤率（cytotoxity index, CI）]=（1-OD_处理_/OD_对照_）×100%。

#### 1.2.3 流式细胞仪检测各组肺癌细胞的细胞周期及凋亡率

继续将上述5组含Fe_3_O_4_磁性纳米粒的培养液调成终浓度为1.5 mg/mL、3.0 mg/mL、4.5 mg/mL和6.0 mg/mL，每瓶3 mL，每组均为3瓶，磁场作用30 min，培养24 h，胰酶脱壁，制成单细胞悬液，75%乙醇后固定后置4 ℃冰箱中过夜，用Rnase A（终浓度为20 μg/mL）消化RNA，37 ℃孵育30 min，流式细胞仪检查各组肺癌细胞的凋亡率和细胞周期的变化。

#### 1.2.4 光学及电子显微镜观察细胞形态

上述5组细胞磁场作用30 min后，继续培养24 h，0.25%胰酶脱壁，加入RPMI-1640 1 mL制成单细胞悬液，细胞贴壁生长24 h，继续培养48 h，光学显微镜下观察细胞形态的变化。然后用0.25%胰酶消化细胞，800 rpm离心5 min、PBS（pH7.4）洗2次、4%预冷戊二醛4 ℃固定过夜，1%的锇酸溶液固定样品1 h-2 h，将经过渗透处理的样品包埋起来，70 ℃加热过夜，在Reichert超薄切片机中切成70 nm-90 nm的薄片，用柠檬酸铅溶液和醋酸双氧铀50%乙醇饱和溶液各染色15 min，透射电镜观察。

### 1.3 统计学处理

利用SPSS 13.0软件包进行方差分析，数据用Mean±SD表示，*P* < 0.05为差异具有统计学意义。

## 2 结果

### 2.1 各组温度的变化

随着磁流体中Fe_3_O_4_浓度的增加，升温越快，峰值温度越高，B组峰值温度为40.1 ℃，C组的峰值温度为42.5 ℃，D组的峰值温度为45.1 ℃，E组峰值温度为49.1 ℃。

### 2.2 人肺癌细胞A549的生长曲线

随着时间的延长，A组肺癌细胞数逐渐增加。12 h内B组和C组细胞增长受到轻度抑制，但培养24 h后肺癌细胞计数逐渐增加；对于D组和E组，随着时间的延长，肺癌细胞增殖受到明显抑制。加温后24 h，A组的细胞计数为（17.9±0.3）×10^4^/ mL，D组的细胞计数为（5.4±0.1）×10^4^/mL，E组的细胞计数为（1.3±0.1）×10^4^/mL，与对照组比较，均有明显差异（*P* < 0.01）；加温后48 h，对照组细胞计数为（46.1± 0.9）×10^4^/mL，D组的细胞计数为（5.9±0.4）×10^4^/mL，与对照组比较，均有明显差异（*P* < 0.01），而加温后48 h E组的细胞全部死亡。具体细胞生长曲线见图 2。

**2:**
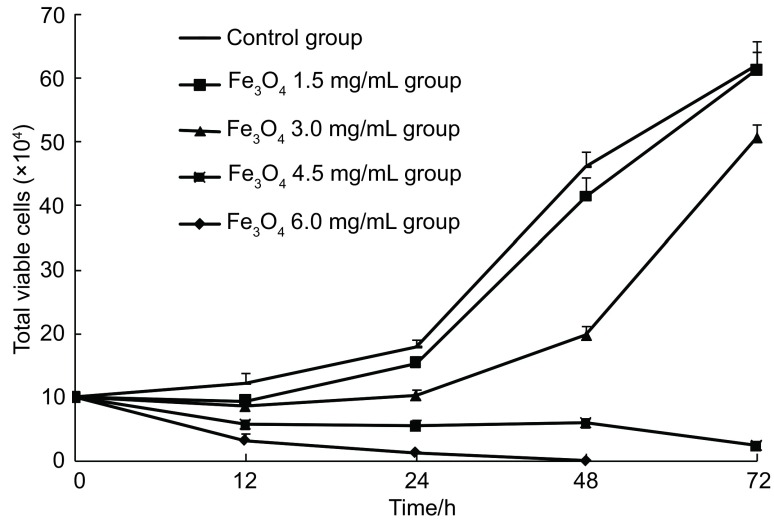
各组人肺癌细胞A549生长曲线比较 The growth curve of cells in different groups

### 2.3 各组人肺癌细胞A549的光密度值及杀伤率

随着磁流体浓度的增加，5个组OD值逐渐变小，CI值逐渐增大，杀伤率增加。A组的OD值为0.17±0.01，B组OD值为0.17± 0.01，C组OD值为0.15±0.02，D组OD值为0.07±0.02，E组OD值为0.05±0.02。

### 2.4 流式细胞仪检测人肺癌细胞A549的凋亡率及细胞周期

流式细胞术检测A组、B组、C组、D组、E组人肺癌细胞A549的凋亡率分别为（2.41±0.20）%、（6.30± 0.80）%、（14.22±1.60）%、（27.06±1.20）%和（49.53 ±3.10）%，与A组比较，有明显差异（*F*=413.243, *P* < 0.001）（图 3）。流式细胞术检测A组、B组、C组、D组、E组人肺癌细胞A549的细胞周期提示，人肺癌细胞A549细胞G_1_期细胞随着Fe_3_O_4_浓度的增加呈现明显减少趋势，S期和G_2_期细胞呈现逐渐增加的趋势；D组与E组的细胞周期间无明显差异（*P*>0.05）（表 1）。

**3:**
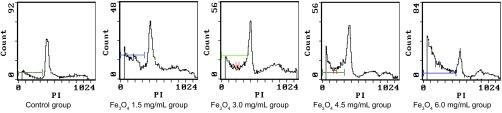
磁流体热疗后各组人肺癌细胞凋亡和周期图 The apoptosis of A549 cells were observed and the cells were arrested at the stage S

**1:** 各组人肺癌细胞A549细胞周期变化 Comparison of cell cycles of A549 cells among different groups

Group	Cell cycle		
	G_0_/G_1_	S	G_2_/M
Control	56.58±1.25	29.62±0.16	13.08±1.06
Fe_3_O_4_ 1.5 mg/mL (40.1 ℃)	55.17±0.78	30.43±1.03	14.41±0.72
Fe_3_O_4_ 3.0 mg/mL (42.5 ℃)	50.15±1.16^※★^	31.83±0.11^※★^	18.01±0.05^※★^
Fe_3_O_4_ 4.5 mg/mL (45.1 ℃)	45.81±0.46^※★^	33.63±0.11^※★^	20.67±0.18^※★^
Fe_3_O_4_ 6.0 mg/mL (49.1 ℃)	46.93±1.06^※★△^	32.76±1.01^※★△^	20.31±1.18^※★△^
Comparison among groups, *F* value of G_0_/G_1_, S and G_2_/M were 26.165, 11.231 and 50.314, *P* < 0.001; compared with control group, ^※^*P* < 0.05; compared with Fe_3_O_4_ 1.5 mg/mL group, ^★^*P* < 0.05; compared with Fe_3_O_4_ 4.5 mg/mL group, ^△^*P*>0.05.			

### 2.5 光学及电子显微镜观察细胞形态

倒置光学显微镜下观察各组细胞形态变化，A组和B组（40.1 ℃）细胞大小基本一致，无细胞破裂，细胞数较多，生长旺盛。C组、D组、E组随着磁流体浓度的升高，正常细胞数逐渐减少，坏死细胞及细胞碎片逐渐增多（图 4）。通过电镜观察发现，热疗后细胞染色质浓集、胞浆空泡化、细胞核裂解，其中由于温度达到49.1 ℃，E组细胞核呈现溶解，正常细胞结构消失，呈坏死表现（图 5）。

**4:**
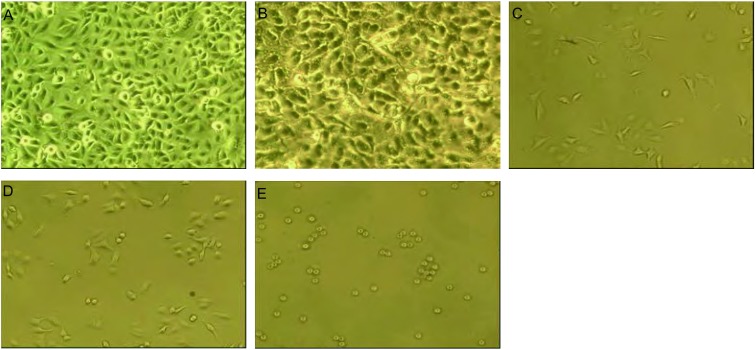
磁流体热疗后各组人肺癌细胞的光镜下形态学改变（×400）。A（Control组）和B（Fe_3_O_4_ 1.5 mg/mL组）：细胞生长形态基本差别不大，呈梭形，分布规则，胞核清晰突出；C：Fe_3_O_4_ 3.0 mg/mL组，细胞开始出现死亡；D：Fe_3_O_4_ 4.5 mg/mL组，随着温度升高，死亡的细胞越多；E：Fe_3_O_4_ 6.0 mg/mL组，细胞全部死亡 Changes in cell morphology were observe by light microscopy (×400). A (Control group) and B (Fe_3_O_4_ 1.5 mg/mL group): The human being carcinoma A549 cells have no evident changes; C (Fe_3_O_4_ 3.0 mg/mL group) and D (Fe_3_O_4_ 4.5 mg/mL group): Most of the human being carcinoma A549 cells were observed to have cell death, karyopyknosis by light microscopy; E (Fe_3_O_4_ 6.0 mg/mL group): All cells were observed to have cell death

**5:**
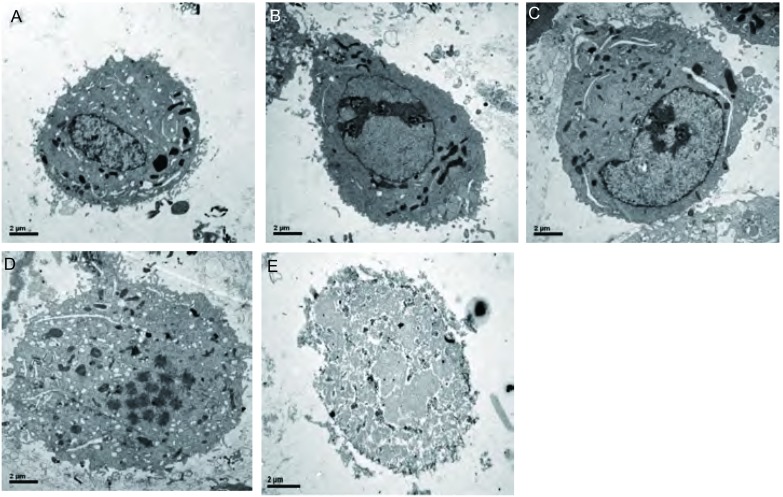
磁流体热疗后各肺癌细胞电镜观察图（×8, 000）。A（Control组）：正常A549肺癌细胞的电镜下表现；B（Fe_3_O_4_ 1.5 mg/mL组）：肺癌细胞热疗后电镜观察无明显改变；C（Fe_3_O_4_ 3.0 mg/mL组）：肺癌细胞热疗呈凋亡样改变，表现为染色体边聚；D（Fe_3_O_4_ 4.5 mg/mL组）：肺癌细胞热疗后表现为核碎裂，也是凋亡的一种形态学表现；E（Fe_3_O_4_ 6.0 mg/mL组）：在49.1 ℃较高温度条件下表现为坏死样改变，细胞正常形态消失，核溶解，大量细胞碎片形成 Changes in cell morphology were observed by electron microscopy (×8, 000). A (Control group): Normal human being carcinoma A549 cell; B (Fe_3_O_4_ 1.5 mg/mL group): A549 cells does not have apparent change; C (Fe_3_O_4_ 3.0 mg/mL group): A549 cells were observed to have margination; D (Fe_3_O_4_ 4.5 mg/mL group): A549 cells were observed to have a form of nuclear fragmentation in apoptosis by electron microscopy; E (Fe_3_O_4_ 6.0 mg/mL group): Necrosis were observed at 49.1 ℃

## 3 讨论

肺癌的发病率和死亡率已居所有癌症之首^[10]^，由于早期肺癌没有明显的临床症状，大部分病例发现时均属晚期，手术效果差。传统的放化疗由于毒副作用大且价格昂贵，多数患者不能耐受；传统的热疗方法如射频、微波、超声等均有加温的特异性差、不能针对某一特定靶区加温的缺陷，大大降低了热疗的效果。因此探索一种治疗效果好、副作用小的热疗方法成为广大从事肺癌研究学者的目标。

磁流体热疗的优点主要是其高度的靶向性和特异性^[11]^，因此它是一种非常有前途的肿瘤治疗方法。在体外，我们探讨了不同温度条件下磁流体热疗对人肺癌细胞的影响。结果发现，磁流体的温度与其浓度呈量效关系，浓度大，峰值温度高，对肺癌细胞的增殖抑制作用越明显。研究发现，4个实验组中，最低浓度B组（1.5 mg/mL）峰值温度为40.1 ℃，未达到热疗的有效范围温度^[11]^，对肺癌细胞的增殖无明显抑制，而C、D、E三组的温度均达到了有效热疗温度，细胞的生长受到抑制。其中最高浓度的E组（6.0 mg/mL）峰值温度为49.1 ℃，人肺癌细胞的生长受到明显的抑制，48 h后细胞几乎全部死亡。这说明可以通过控制磁流体的剂量来实现热疗的控温^[12]^，从而选择有效的温度来杀死肿瘤细胞，达到治疗的目的。

研究证实，热疗可以通过直接物理作用造成正常细胞和肿瘤细胞的凋亡和坏死^[13]^，且温度越高，凋亡越明显^[14]^。凋亡是一种细胞主动自杀的过程，是细胞死亡的一种特定形式，凋亡有其自身的特征性改变，包括形成DNA碎片、染色质边集、胞浆空泡化、细胞皱缩以及形成凋亡小体等^[15, 16]^，研究^[17]^发现，肿瘤细胞在42.0 ℃的条件下就可以凋亡。我们的实验结果发现，浓度≥3.0 mg/mL时，峰值温度≥42.5 ℃，细胞增殖开始受到抑制，温度越高，抑制越明显。电镜下可见明显的凋亡改变。流式细胞术检测发现，随着温度的增高，凋亡率越高。其中E组（6.0 mg/mL, 49.1 ℃）的肺癌细胞48 h后几乎全部凋亡、坏死，说明温度越高凋亡越明显，治疗效果越好。

真核细胞周期分为有丝分裂期和间期，细胞的生长、分裂依次经过G_1_期、S期、G_2_期、M期，至于热疗后对细胞周期各期的阻滞均有报道^[18-22]^，我们用流式细胞仪测定热疗后人肺癌A549细胞周期的变化，结果表明磁流体热疗后细胞周期发生了明显的改变，G_1_期细胞减少，S期和G_2_期细胞增多。说明磁流体热疗后人肺癌A549细胞出现S期阻滞。S期是细胞周期进程中一个十分重要的阶段，此阶段主要是大量的DNA复制过程，同时也合成组蛋白和非组蛋白，最后完成染色体的复制。因此S期的阻滞对DNA的复制产生了巨大的影响，进而导致细胞凋亡。

尽管学者们对热疗治疗肿瘤的机理做了大量的研究，其中诱导凋亡、导致坏死、阻滞细胞周期是目前大家比较认可的机制之一，但确切的机制尚无定论，磁流体热疗的确切机制同样有待于进一步研究。本实验通过不同浓度的Fe_3_O_4_磁流体在交变磁场作用下在体外对肺癌细胞的影响的研究，初步证实磁流体热疗可以明显抑制人肺癌A549细胞的增殖，诱导其凋亡、导致坏死、阻滞细胞于S期，是磁流体热疗对人肺癌研究的一个切入点，其结果必将为磁流体热疗在肺癌领域的进一步研究打下良好的基础。
